# Multiple-Inputs Convolutional Neural Network for COVID-19 Classification and Critical Region Screening From Chest X-ray Radiographs: Model Development and Performance Evaluation

**DOI:** 10.2196/36660

**Published:** 2022-10-04

**Authors:** Zhongqiang Li, Zheng Li, Luke Yao, Qing Chen, Jian Zhang, Xin Li, Ji-Ming Feng, Yanping Li, Jian Xu

**Affiliations:** 1 Division of Electrical and Computer Engineering College of Engineering Louisiana State University Baton Rouge, LA United States; 2 Division of Computer Science and Engineering College of Engineering Louisiana State University Baton Rouge, LA United States; 3 Department of Visualization Texas A & M University College Station, TX United States; 4 Department of Comparative Biomedical Science School of Veterinary Medicine Louisiana State University Baton Rouge, LA United States; 5 School of Environment and Sustainability University of Saskatchewan Saskatoon, SK Canada

**Keywords:** COVID-19, chest X-ray radiography, multiple-inputs convolutional neural network, screening critical COVID regions

## Abstract

**Background:**

The COVID-19 pandemic is becoming one of the largest, unprecedented health crises, and chest X-ray radiography (CXR) plays a vital role in diagnosing COVID-19. However, extracting and finding useful image features from CXRs demand a heavy workload for radiologists.

**Objective:**

The aim of this study was to design a novel multiple-inputs (MI) convolutional neural network (CNN) for the classification of COVID-19 and extraction of critical regions from CXRs. We also investigated the effect of the number of inputs on the performance of our new MI-CNN model.

**Methods:**

A total of 6205 CXR images (including 3021 COVID-19 CXRs and 3184 normal CXRs) were used to test our MI-CNN models. CXRs could be evenly segmented into different numbers (2, 4, and 16) of individual regions. Each region could individually serve as one of the MI-CNN inputs. The CNN features of these MI-CNN inputs would then be fused for COVID-19 classification. More importantly, the contributions of each CXR region could be evaluated through assessing the number of images that were accurately classified by their corresponding regions in the testing data sets.

**Results:**

In both the whole-image and left- and right-lung region of interest (LR-ROI) data sets, MI-CNNs demonstrated good efficiency for COVID-19 classification. In particular, MI-CNNs with more inputs (2-, 4-, and 16-input MI-CNNs) had better efficiency in recognizing COVID-19 CXRs than the 1-input CNN. Compared to the whole-image data sets, the efficiency of LR-ROI data sets showed approximately 4% lower accuracy, sensitivity, specificity, and precision (over 91%). In considering the contributions of each region, one of the possible reasons for this reduced performance was that nonlung regions (eg, region 16) provided false-positive contributions to COVID-19 classification. The MI-CNN with the LR-ROI data set could provide a more accurate evaluation of the contribution of each region and COVID-19 classification. Additionally, the right-lung regions had higher contributions to the classification of COVID-19 CXRs, whereas the left-lung regions had higher contributions to identifying normal CXRs.

**Conclusions:**

Overall, MI-CNNs could achieve higher accuracy with an increasing number of inputs (eg, 16-input MI-CNN). This approach could assist radiologists in identifying COVID-19 CXRs and in screening the critical regions related to COVID-19 classifications.

## Introduction

### Background

In early 2020, COVID-19 was officially announced as a pandemic by the World Health Organization (WHO), which rapidly spread to become one of the largest unprecedented health crises worldwide [1]. To date, the COVID-19 pandemic has heavily impacted the global economy and threatened many people’s lives [1]. According to the latest WHO reports, by July 2021, 190,671,330 people have been confirmed to have COVID-19, contributing to 4,098,758 deaths. In the United States, there have been 33,741,532 confirmed cases with 603,880 deaths [2]. At the time of writing, the United States, India, and Brazil have the highest numbers of confirmed cases globally, followed by France, Russia, Turkey, and the United Kingdom [2].

Luckily, several types of COVID-19 vaccines have been rapidly and accurately developed, such as Pfizer-BioNTech, Moderna, Johnson & Johnson’s Janssen, and others, which are reaching an increasing number of populations worldwide [3]. For instance, by July 2021, 3,436,534,998 vaccine doses had been administered worldwide and 341,759,270 vaccine doses had been administered in the United States [2]. Unfortunately, the crisis of COVID-19 remains severe, primarily since the Delta variant of SARS-CoV-2 was first identified in December 2020 in India, followed by the second large wave in the country. This new variant quickly spread to more than 92 countries to become the dominant viral COVID-19 strain in the world [4]. Moreover, a more recent variant of SARS-CoV-2, Omicron, was reported in December 2021 and spread globally thereafter [5-7].

Currently, polymerase chain reaction (PCR), especially real-time reverse-transcription-PCR (RT-PCR), is considered the gold standard for diagnosing COVID-19. However, this method has many problems such as being time-consuming or requiring specialized personnel and laboratories [8,9]. In addition, medical imaging such as chest X-ray radiography (CXR), chest computed tomography (CT), and magnetic resonance imaging also serves as an important alternative method for COVID-19 diagnosis [1,8,10]. CXR is the imaging technique that was first used to diagnose COVID-19 and continues to play an important role in clinical diagnosis [11-16].

A chest CT scan could be more sensitive than CXR for the diagnosis of COVID-19; however, some significant issues hinder its use, such as high costs, time-intensive processes to scan a single patient, high levels of ionizing radiation, and limited access in some hospitals or health centers [8-16]. Therefore, CXR remains an affordable imaging technique that is widely used to diagnose COVID-19 with a much lower radiation dose [17]. In addition, in clinical practice, the RT-PCR test is often combined with a CXR examination to reduce the false negatives, and to assess the extent and severity of the disease [8,9].

### Prior Work

In some conditions, extracting and finding useful image features from CXRs impose a heavy workload for radiologists [9,15,18]. In recent years, deep learning has become one of the most popular research topics in image classification, identification, and segmentation [8,19-24]. Compared to conventional approaches of image analysis, deep-learning methods usually have better efficiency in extracting image features since they do not require human supervision to determine the critical image features. The convolutional neural network (CNN) is one of the most representative examples of deep learning for learning and recognizing specific image features [8,19,20,25]. Therefore, integrating an efficient CNN architecture into diagnostic systems would help to reduce the workload of radiologists, while increasing the reliability of the result and enabling quantitative analysis [20]. To date, several CNN models have been reported to differentiate COVID-19 cases from other (non-COVID-19) cases with CXR, including GoogleNet, ResNet50, VGG19, MobileNetV2, and Inception. Most of these models could achieve very high accuracy (up to 99%) in the classification of COVID-19 [11,13-16,18,26-31]. Thus, deep learning with CXRs could be a valuable method to identify COVID-19.

In most of these previous studies, the CNN models were trained with whole-image CXRs as a single input for the classification [9,11,13-16,18,26-31]. Other studies also attempted to develop new CNN models that accept multiple inputs; such multiple-input CNNs (MI-CNN) could effectively improve the classification accuracy and demonstrated better performance than single-input CNNs [32-34]. Because an MI-CNN could provide different features, fusing these network features together could improve the accuracy of the entire system [34]. To date, MI-CNNs have been applied in the fields of facial expression and gender recognition [33,34] or flower grading [35].

However, most of the MI-CNN models developed in previous studies used whole images as at least one of the CNN inputs, and the prefeatured images were used as the other inputs [33,34]. To our best knowledge, few studies have reported using MI-CNNs to detect and analyze COVID-19 CXRs. In addition, some of the obtained features can allow the network to determine the correct result, while other features can also cause serious misjudgment [34]. Thus, evaluation of feature contribution and removal of negative CNN features are critical steps toward increasing the reliability of disease diagnosis. However, few studies have explored the feasibility of using an MI-CNN to extract important image regions and exclude the contribution of the irrelevant features for the classification of COVID-19.

In this study, we developed a novel COVID-19 classification strategy with MI-CNN models. CXR images could be evenly segmented into different regions, and each MI-CNN input could process only one part of the COVID-19 CXRs. Furthermore, MI-CNNs could screen the critical regions for the classification of COVID-19 CXRs and exclude irrelevant image regions that falsely contribute to the COVID-19 classification.

## Methods

### COVID-19 and Normal CXR Image Data Sets

In this study, 6205 CXR images (including 3021 COVID-19 CXRs and 3184 normal CXRs) were obtained from previous reports [14,36,37]. All of the CXR images were resized to 320×320 pixels to obtain 16 image segmentations. All CXR images were used in the original PNG format without any modification.

### Ethics Approval

The ethics review was waived due to the use of secondary publicly available data, along with a lack of manipulation or intervention of human subjects, as determined by the Louisiana State University Institutional Review Board.

### Design of the MI-CNN Architecture

**Figure 1:**
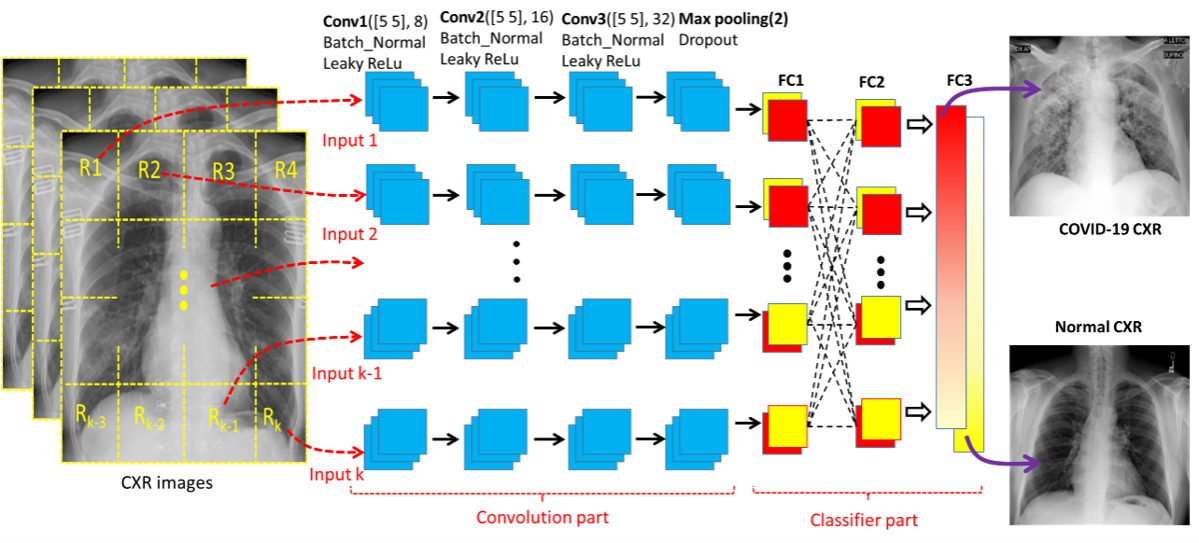
Schematic diagram of the multiple-input convolutional neural network (MI-CNN) architecture. There are two parts in the MI-CNN: the convolution part and classifier part. The convolution part consists of up to 16 MI-CNN inputs (depending on the type of MI-CNN), and each MI-CNN input has three convolutional sections and one max-pooling layer. The classifier part is composed of three fully connected (FC) layers. CXR: chest X-ray radiograph; ReLu: rectified linear unit.

Figure 1 provides a schematic diagram of the MI-CNN architecture, which is composed of the convolution part and classifier part. Each CXR was evenly segmented into 2, 4, or 16 different regions, and each type of image segmentation was loaded into the corresponding MI-CNN model (2-input, 4-input, and 16-input MI-CNNs). For the single-input CNN, the whole CXR image was directly loaded into the model. For the convolution part, each MI-CNN input had three convolutional sections. Each convolutional section included one 2D convolutional layer, one batch normalization (Batch_Normal), and one leaky rectified linear unit (ReLu) layer. All three 2D convolutional layers were set to a (5, 5) filter size. The filter number was set to 8 for the first 2D convolutional layer (Conv1), 16 for the second 2D convolutional layer (Conv2), and 32 for the third 2D convolutional layer (Conv3). A one max-pooling layer was used after the three convolutional sections.

There were three fully connected (FC) layers for the classifier part: the first FC (FC1) was set to receive the outputs from each MI-CNN input, FC2 was used to fully connect all of the FC1 outputs from all MI-CNN inputs, and FC3 was used to determine the CXR category (COVID-19 or normal). Accuracy, sensitivity, specificity, and precision were calculated for model performance evaluation.

### Segmentation of Lung Regions Through the Most Strongly Activated Convolutional Layer

**Figure 2:**
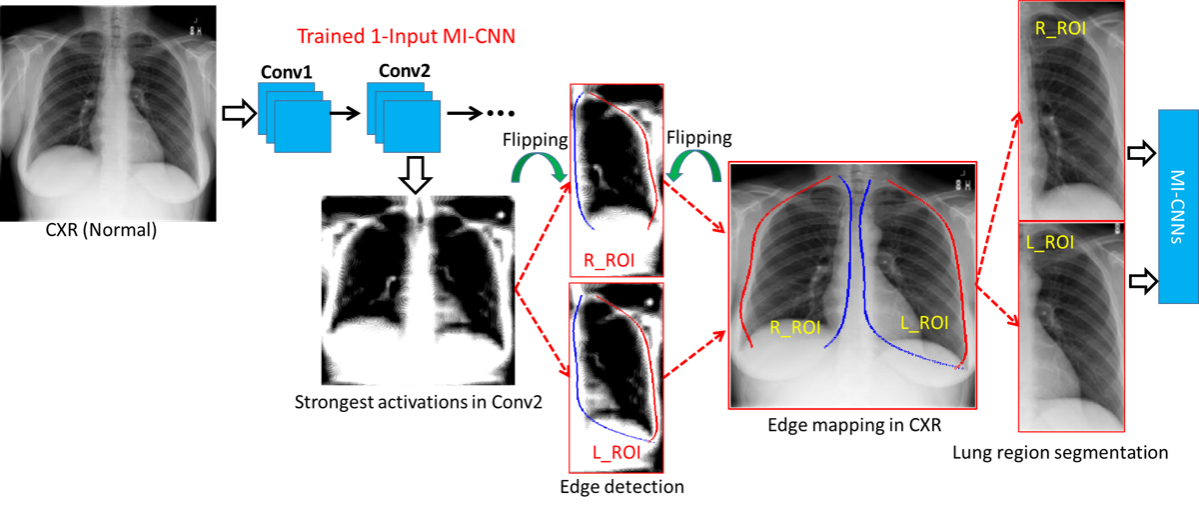
Schematic diagram of the regions of interest for the right and left lung (R_ROI and L_ROI, respectively) based on the strongest activation of max-pooling layers in the 1-Input convolutional neural network (CNN) model. Conv: convolutional layer; CXR: chest X-ray radiography; MI-CNN: multiple-input convolutional neural network.

The crucial steps in the automatic analysis of CXRs are accurate lung boundaries detection and their classification as normal or abnormal [17]. Segmentation of lung boundaries in medical imaging allows for disease identification, including for the detection of COVID-19 [17]. In the single-input CNN, the strongest activations of the second convolutional layer could yield the best profile of the lung regions, as shown in Figure 2. To extract both the left-lung region of interest (ROI) and the right-lung ROI, the CXR was first divided into two even parts and then the left part of the CXRs was flipped over horizontally.

Since the lung regions are relatively darker than the surrounding anatomical structures (the white regions), the edges of the left and right lung regions could be determined by the starting (blue lines) and end (red lines) points in each column. The coordinates of the lung edges were then projected onto the original CXRs. However, for some CXRs from patients at severe COVID-19 stages, it can be challenging to identify the lung regions from surrounding regions. Therefore, a rectangular region with minimum and maximum coordinates of the lung edges was used to crop the whole lung regions.

### Screening Critical Regions for COVID-19 Classification

**Figure 3:**
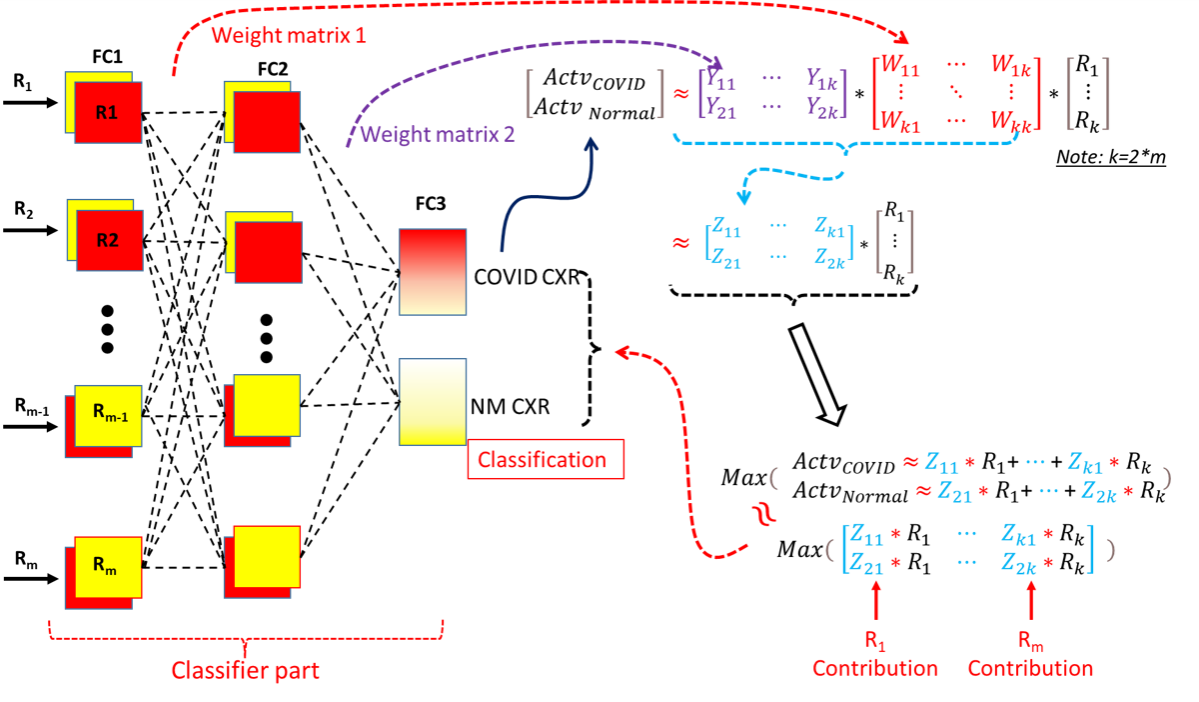
Schematic diagram of the classifier in the multiple-input convolutional neural network (MI-CNN) and screening of critical regions that classify COVID-19 and normal (NM) chest X-ray radiographs (CXRs). FC: fully connected layer; R: matrix between MI-CNN inputs; W: weight matrix of the second fully connected layer (FC2); Y: weight matrix of the last fully connected layer (FC3); Z: product of the Y and W matrices.

Although deep-learning approaches have been widely applied in analyses of medical images, few studies have reported the use of MI-CNN models to analyze the contributions of the critical ROIs and exclude the false contributions of regions that are irrelevant for the classification of diseases. To find the critical regions and exclude the irrelevant regions for the classification of COVID-19 CXRs, we explored the relationship between the outputs (*R* matrix) of each convolutional branch (also serving as the inputs of the classifier part) and the final activations (FC3 layer) of the classifier part, as shown in Figure 3.

The approximate relationship between FC1 and FC2 activations could be found through the weight matrix of the FC2 layer (*W* matrix in Figure 3). The weight matrix of the FC3 layer (*Y* matrix in Figure 3) could provide an approximate relationship between FC2 and FC3 activations. Therefore, the relationship between MI-CNN inputs (*R* matrix) and the final classification (FC3 activations) could be approximately evaluated through the matrix product (*Z* matrix) of the *W* and *Y* weight matrices. Furthermore, the final classification of COVID-19 or normal CXR was approximately determined by the maximum values of FC3 activations. The classification could then be approximately determined by the maximum elements of the element-wise multiplication between the *Z* matrix and *R* matrix (Figure 3).

Regarding the region contributions, the correctly classified images in the testing data sets were grouped with the labels of the corresponding regions (R1 to R16) that gave the maximum elements of element-wise multiplication (*Z* and *R* matrices). The region contributions could then be evaluated according to the percentages of the correctly classified images of each MI-CNN input.

All analyses were conducted with MATLAB R2020b (MathWorks Inc) on an HP Z2620 Workstation computer with a NVIDIA Tesla K80 GPU Accelerator.

## Results

### Evaluation of MI-CNNs With Different Inputs for Discrimination of COVID-19 and Normal CXRs

**Figure 4:**
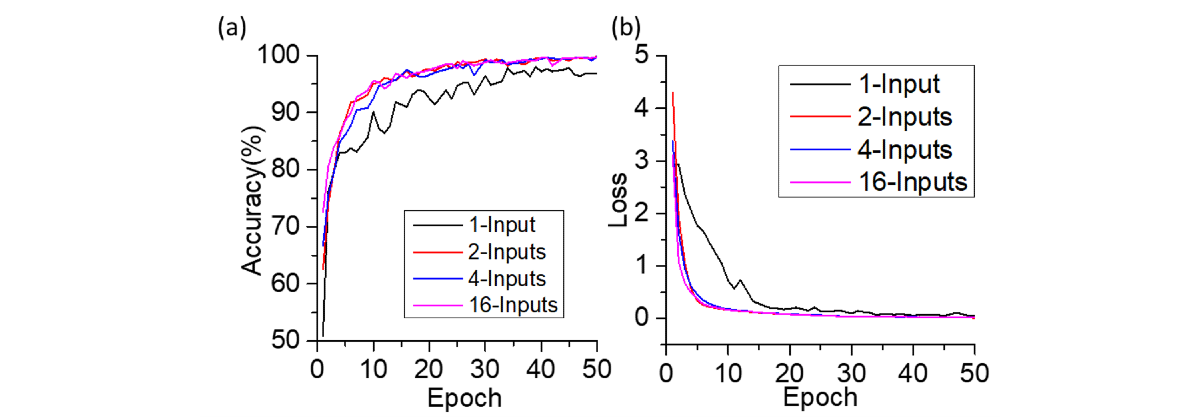
(a) Accuracy-epoch and (b) loss-epoch curves of the 1-input convolutional neural network (CNN) and 2-, 4-, and 16-input CNNs. Each curve represents the average of 5 five-fold cross-validation; the learning rate was 0.01 in all cases.

To evaluate the performance of the single-input CNN and MI-CNNs with 2, 4, and 16 inputs, 90% of the CXR data sets were used for training and the rest (10%) were used for testing. Five-fold cross-validation was used for all MI-CNN models. Figure 4 shows the training accuracy and loss curves of the 1-, 2-, 4-, and 16-input MI-CNNs, and each training had 50 epochs with a 0.01 learning rate. Throughout the 50-epochs training, the three MI-CNNs had higher training accuracy than the 1-input CNN. In addition, the 1-input CNN had much higher loss at the beginning of the training (with 20 epochs) than the MI-CNNs, but showed similar loss to that of the MI-CNNs until 35 epochs.

After 50 epochs, there was an approximate 0.02 training loss for the MI-CNNs and 0.05 loss for the 1-input CNN. The MI-CNNs also had approximately 3% higher accuracy than the 1-input CNN (~99% vs 96%). In addition, at the beginning of the training curves, MI-CNNs showed higher training accuracy, which was 50.94% for the 1-input CNN, 62.53% for the 2-inputs MI-CNN, 66.66% for the 4-inputs MI-CNN, and 72.60% for the 16-inputs MI-CNN.

**Figure 5:**
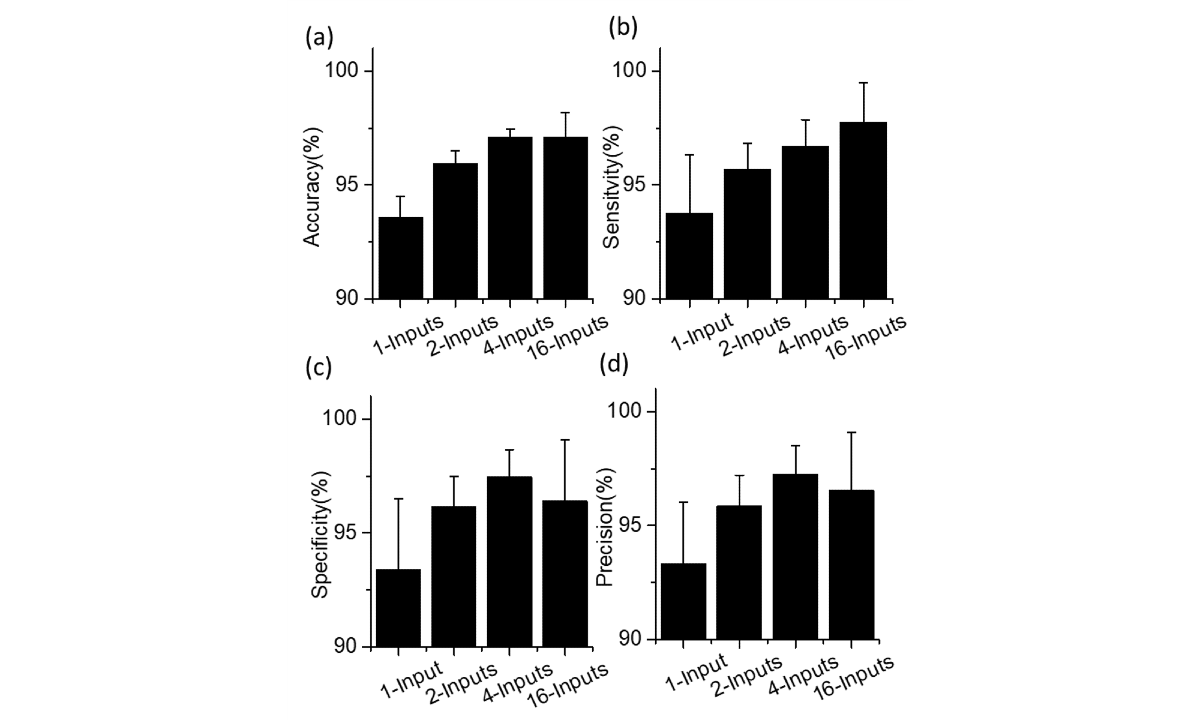
(a) Accuracy, (b) sensitivity, (c) specificity, and (d) precision of the of 1-input convolutional neural network (CNN), and the 2-, 4-, and 16-input CNNs.

Regarding the testing evaluations (Figure 5), all MI-CNNs exhibited good performance in the classification of COVID-19 and normal CXRs, which could achieve over 93% accuracy, sensitivity, specificity, and precision. Similar to the training accuracy and loss, MI-CNNs with more than 2 inputs also exhibited better classification efficiency than the 1-input CNN. For instance, the MI-CNNs usually had over 95% accuracy, sensitivity, specificity, and precision, whereas these metrics only reached around 93% for the 1-input CNN under the same conditions.

However, for MI-CNNs, the testing performance increased with more inputs, in which the 16-inputs MI-CNN showed the best classification of COVID-19 CXRs and exhibited the highest accuracy (up to a mean of 97.10%, SD 1.08%) and sensitivity (up to a mean of 97.77%, SD 1.71%); however, there was only a minimal difference in the specificity and precision between the 2-input and 16-input MI-CNNs (approximately 1%-2% smaller than those of the 4-inputs MI-CNN).

**Figure 6:**
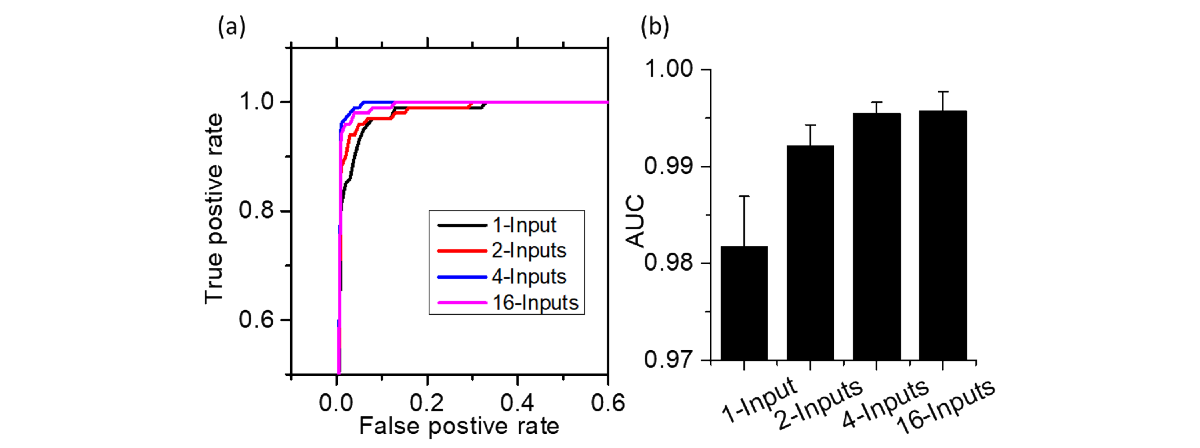
Receiver operating characteristic curves (a) and area under the curve (AUC) values (b) of the 1-input convolutional neural network (CNN), and 2-, 4-, and 16-input CNNs.

As shown in the receiver operating characteristic (ROC) curves in Figure 6, all MI-CNNs with different inputs had good efficiency in the classification of COVID-19 and normal CXR, with the 4-inputs MI-CNN showing the best performance (Figure 6a). Similar to the testing data sets, the MI-CNNs had an area under the ROC curve (AUC) value of 0.98 for all inputs. However, the MI-CNNs had much higher AUC values (over 0.99) than that of the 1-input CNN (mean 0.982, SD 0.005), and the 4-inputs and 16-input MI-CNNs had the largest AUC values overall (0.995) (Figure 6b).

### Classification of COVID-19 and Normal CXRs With Left- and Right-Lung ROI Data Sets

**Figure 7:**
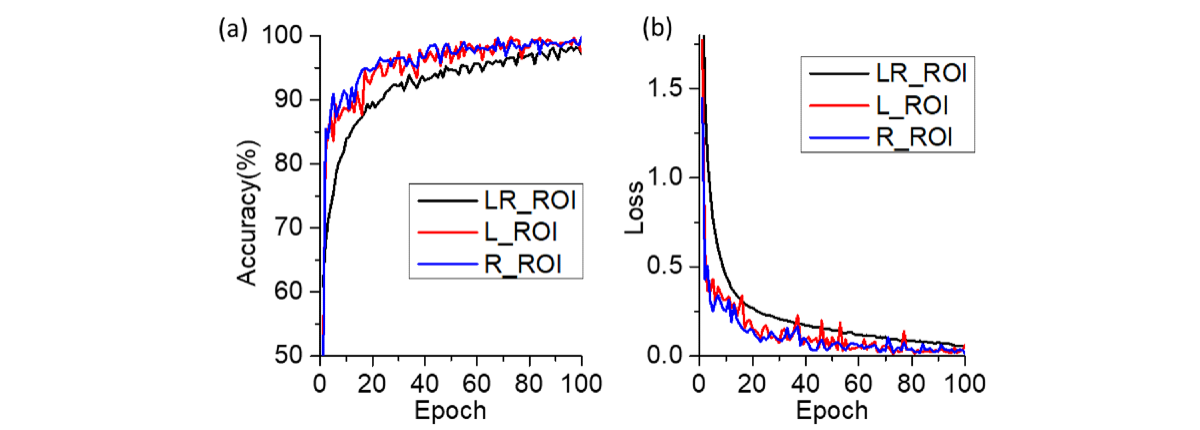
(a) Accuracy-epoch and (b) loss-epoch curves of the 1-input convolutional neural network (CNN) and 2-inputs CNN with the left-lung region of interest (L-ROI), right-lung region of interest (R-ROI), and left and right lung region of interest (LR-ROI) data sets. Each curve represents the average of five replicates; the learning rate was 0.001.

Figure 7 indicates the training accuracy epoch and loss epoch of the ROI data sets with the 1-input CNN and 2-inputs MI-CNN. Left- and right-lung ROI curves were obtained from the corresponding data sets of left or right lung regions, which were trained with the 1-input CNN. The LR-ROI curves were obtained from the left and right data sets, serving as the two inputs of the 2-inputs MI-CNN. All of these models were run with 100 epochs and a 0.001 learning rate, and were repeated five times.

From Figure 7, it can be seen that the 1-input CNN (both left- and right-lung ROIs) exhibited a higher accuracy curve and lower loss curve than the 2-inputs MI-CNN with the LR-ROI data set; however, all three methods showed similar accuracy (~97.0%) and loss (~0.05) at the end of the training. The LR-ROI data set resulted in approximately 15% higher accuracy than that of the left and right ROI data sets at the beginning of the training.

**Figure 8:**
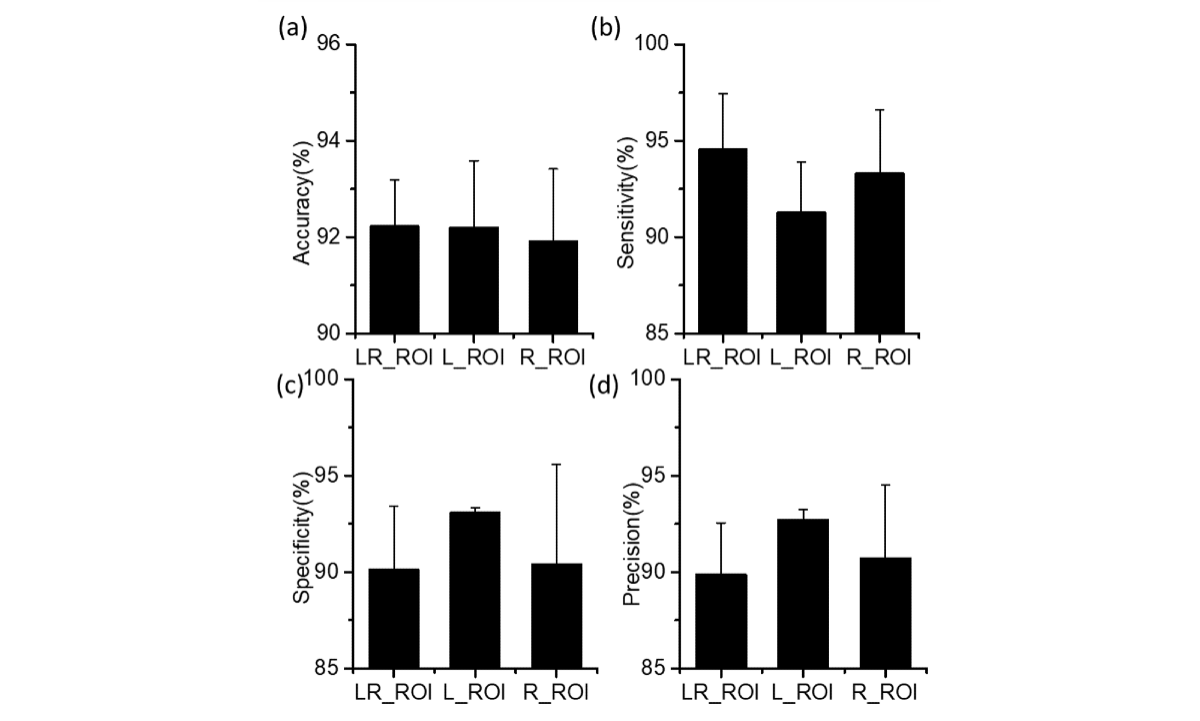
(a) Accuracy, (b) sensitivity, (c) specificity, and (d) precision of the 1-input convolutional neural network (CNN) with the left-lung region of interest (L-ROI) and right-lung region of interest (R-ROI), and the 2-inputs CNNs with the combined left- and right-lung region of interest (LR-ROI) data set.

For the LR-ROI testing data sets (Figure 8), MI-CNNs showed good efficiency for the classification of COVID-19 CXRs, although the accuracy, sensitivity, specificity, and precision were slightly lower than those of the whole-image data sets (but still above 90%). There was almost no difference in the accuracy between the 1-input CNN and 2-inputs MI-CNN in the accuracy (~92%) for all three data sets (Figure 8a). LR-ROI showed the largest sensitivity (up to a mean of 94.55%, SD 2.90%), followed by the right ROI (93.30%, SD 3.27%) and then the left ROI with a slightly lower value (91.27%, SD 2.64%). By contrast, the left-lung ROI data set had larger specificity (mean 93.09%, SD 0.23%) and precision (mean 92.71%, SD 0.52%) than those of the right-lung ROI and LR-ROI (both approximately 90%).

Therefore, the three ROIs showed similar efficiency (similar accuracy) in classifying COVID-19 CXRs. Compared to the left-lung ROI method, higher sensitivity with the LR-ROI and right-lung ROI data sets indicated that the two CNN models (especially the 2-inputs MI-CNN) had better capability to identify COVID-19 CXRs from the normal CXRs correctly. Left-lung ROI had a lower probability of falsely recognizing normal CXRs as COVID-19 CXRs. Overall, LR-ROI and right-lung ROI had better efficiency in detecting COVID-19 CXRs, while the left-lung ROI method was better for identifying normal CXRs.

**Figure 9:**
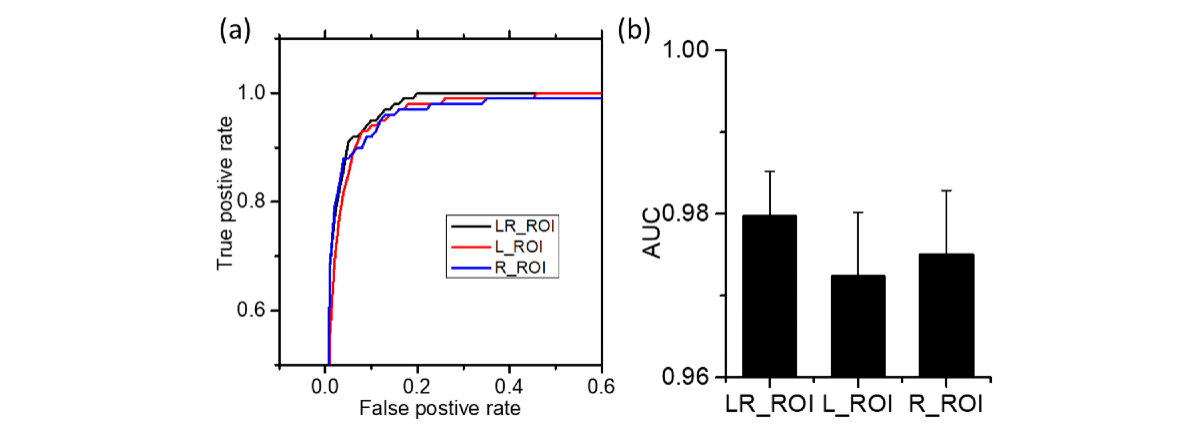
Receiver operating characteristic curves (a) and area under the curve (AUC) values (b) of the 1-input convolutional neural network (CNN) with the left-lung region of interest (L-ROI) and right-lung region of interest (R-ROI), and the 2-inputs CNN with the left- and right-lung region of interest (LR-ROI).

Based on the ROC curve, the 2-inputs MI-CNN with the LR-ROI data sets also showed relatively better performance than the 1-input CNN (left- or right-lung ROI) in classifying COVID-19 and normal CXRs, given its larger AUC value (mean 0.980, SD 0.005), as shown in Figure 9. In the 1-input CNN, the right-lung ROI data set showed better efficiency (mean AUC 0.975, SD 0.008) in identifying COVID-19 CXRs than the left-lung ROI (mean AUC 0.972, SD 0.008), as shown in Figure 9b.

In addition, LR-ROI data sets were also evaluated using the 4-input and 16-input MI-CNNs (see Figures S1 and S2 in Multimedia Appendix 1). The results showed almost no difference among different inputs of MI-CNNs, although the 2-inputs model had much higher sensitivity (mean 94.55%, SD 2.90%) than that of the 4-input (mean 92.72%, SD 4.37%) and 16-input (mean 93.42%, SD 2.25%) MI-CNNs.

### Screening of Critical Regions for COVID-19 Identification Using MI-CNNs

Regarding the region contributions, 90% of the whole-image and LR-ROI data sets were trained under the same training conditions as shown in Figure 4 and Figure 7, but repeated 10 times. The percentages of the correctly classified images from each image region were then calculated following the procedures outlined in Figure 3.

**Figure 10:**
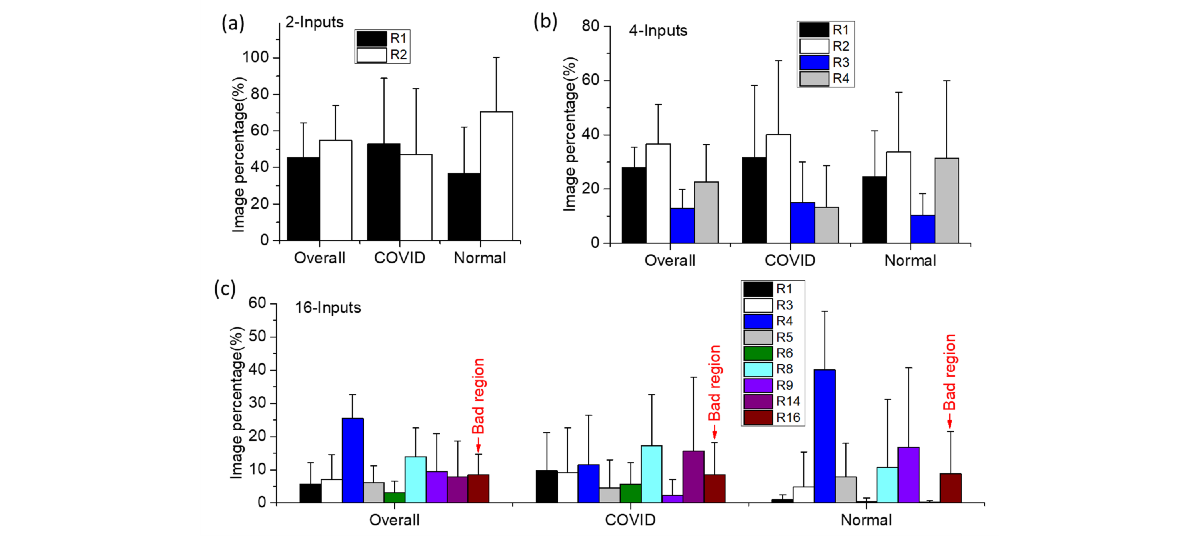
Screening and evaluating the critical regions (R1-R16) of the whole-image data sets through the initial inputs and final activations of the classifier part of multiple-input convolutional neural networks (MI-CNNs): (a) 2-input MI-CNN; (b) 4-input MI-CNN; (c) 16-input MI-CNN.

**Figure 11:**
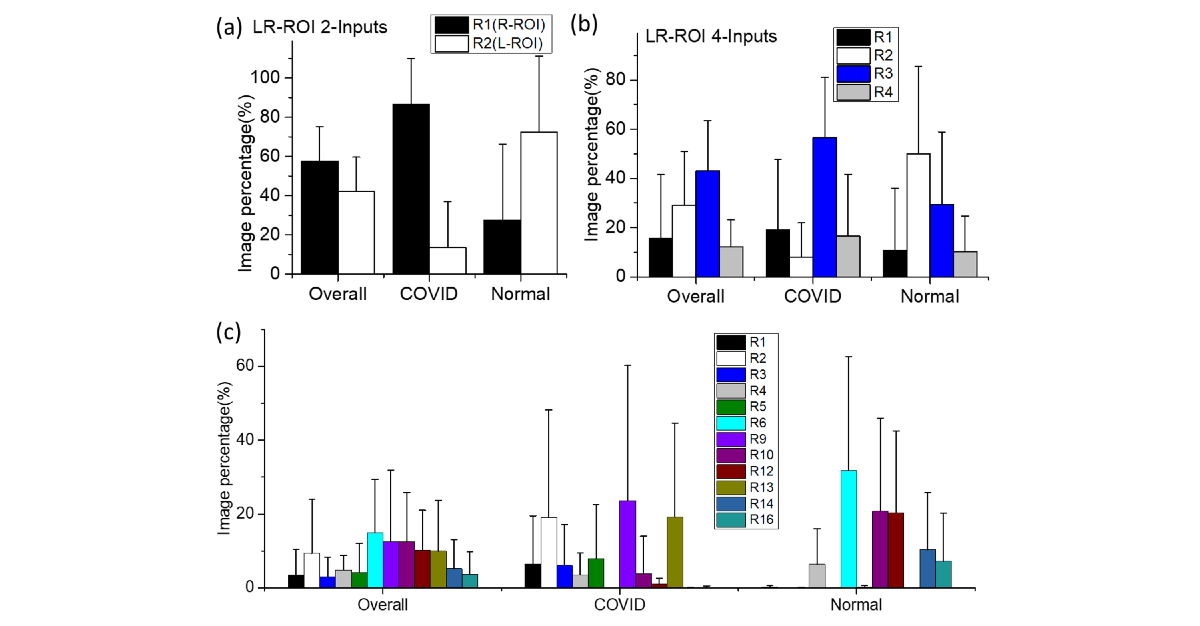
Screening and evaluation of the critical regions of the lung imaging data sets (R1-R16) through the initial inputs and final activations of the classifier part of multiple-input convolutional neural networks (MI-CNNs) with more than two inputs: (a) 2-inputs MI-CNN; (b) 4-inputs MI-CNN; (c) 16-inputs MI-CNN. L-ROI: left-lung region of interest; LR-ROI: left- and right-lung region of interest; R-ROI: right-lung region of interest.

For the 2-inputs MI-CNN, both the whole-image and LR-ROI data sets showed that more COVID-19 CXRs were classified by the R1 regions (right-lung ROI), while more normal CXRs were recognized by the R2 regions (left-lung ROI) (Figures 10a and 11a). However, over 86.5% of COVID-19 CXRs were identified through the R1 regions in LR-ROI data sets, while only 13.5% were identified through the R2 regions. Moreover, normal CXRs showed slight changes in R1 (~30%) and R2 (~70%) between the whole-image and LR-ROI data sets.

Compared to the 2-inputs MI-CNN, all four regions contributed to the COVID-19 classification in the 4-inputs MI-CNN. In the whole-image data sets, R2 had the largest contributions to both COVID-19 and normal CXRs, whereas R3 had the lowest contributions. However, R4 regions showed the greatest difference in the classification of COVID-19 and normal images, which was approximately 35% in normal CRXs but only 10% in COVID-19 CRXs (Figure 10b). In the LR-ROI data sets, COVID-19 had the largest image percentage in the R3 regions (up to 60%) and the lowest image percentage (only 10%) in R2 regions (Figure 11b). In normal CXRs, the largest image percentage was found in R2 (up to 50%, the lowest region for COVID-19), followed by R3 (up to 30%) (Figure 11b).

In the 16-inputs MI-CNN, the critical regions became more obvious because smaller regions were used as MI-CNN inputs. From the whole-image data sets in Figure 10c, R1 regions had the largest contributions in COVID-19 CXRs, accounting for approximately 22% of the total accurately classified COVID-19 images. R6 had the second-largest contribution (accounting for approximately 15% of the correctly classified images). Compared to COVID-19 CXRs, the greatest difference in normal CXRs was found in the R9 regions (up to 20% vs ~3% in COVID-19 images), and R4 regions had the largest contributions (up to 27%) (Figure 10c).

In LR-ROI data sets, the critical regions and irrelevant regions become more clear. In COVID-19 CRXs, significant regions could be found in the R1, R2, R5, and R9 regions, especially R5 accounting for approximately 35%. These regions had almost no contribution in normal CXRs, whereas the greatest critical regions in normal CXRs were R10, R12, and R14, which were much higher (with each contribution reaching up to 20%) than other regions. These regions had almost no contributions to COVID-19 classification.

**Figure 12:**
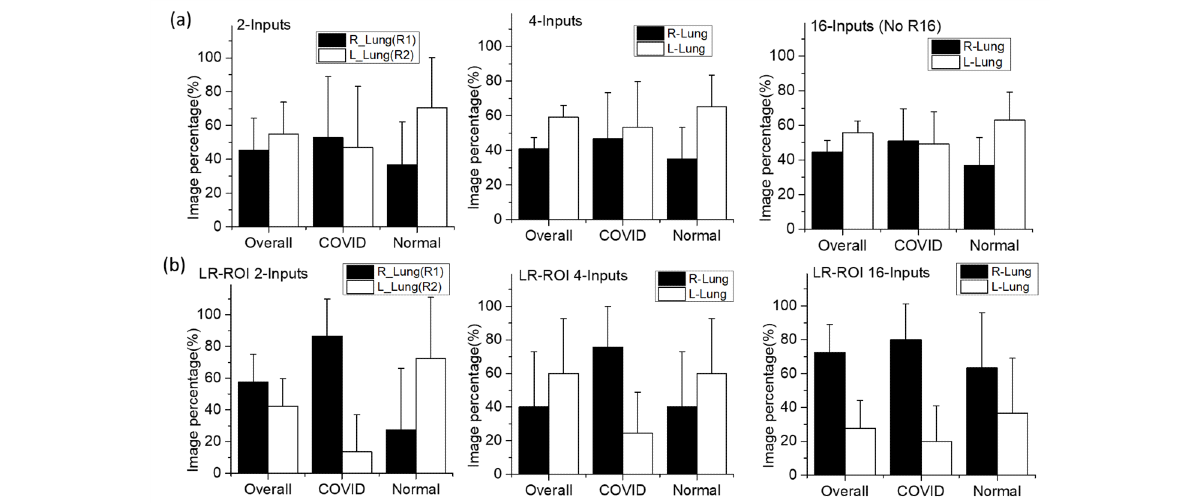
Contributions of the left-lung regions (L–Lung) and right-lung regions (R–Lung) to classifying COVID-19 chest X-ray radiographs (CXR) using multiple-input convolutional neural networks. (a) Whole-image CXR data sets; (b) left- and right-lung region of interest (LR-ROI) data sets.

From the 16-inputs MI-CNN, nonlung regions were found to play critical roles in classifying COVID-19 (eg, R16 contributed up to 15% of testing images). When combining the left- and right-lung regions in 4- and 16-input MI-CNNs, the left lung had a greater contribution to the classification of both COVID-19 and normal CXRs (Figure 12a), which was not consistent with the results for the 2-inputs MI-CNN (Figure 12a). However, if removing the nonlung region R16, the right lung had a greater contribution (approximately 60% of classified testing images) in the classification of COVID-19 CXRs. In comparison, a greater contribution (also approximately 60% of classified testing images) was found from the left lung to classify normal CXRs (Figure 12b). Thus, it seems that COVID-19 CXRs could be more efficiently classified from the right-lung data sets, while normal CXRs were much more easily found through the left-lung data sets. From the color mapping to CXRs shown in Figure S4 of Multimedia Appendix 1, most of the critical regions for the classification of COVID-19 CXRs were distributed in the right-lung regions, while those for normal CXRs were in the left-lung regions.

### Visualization of the CNN Features From the Max-Pooling Layers in MI-CNNs With Different Inputs

**Figure 13:**
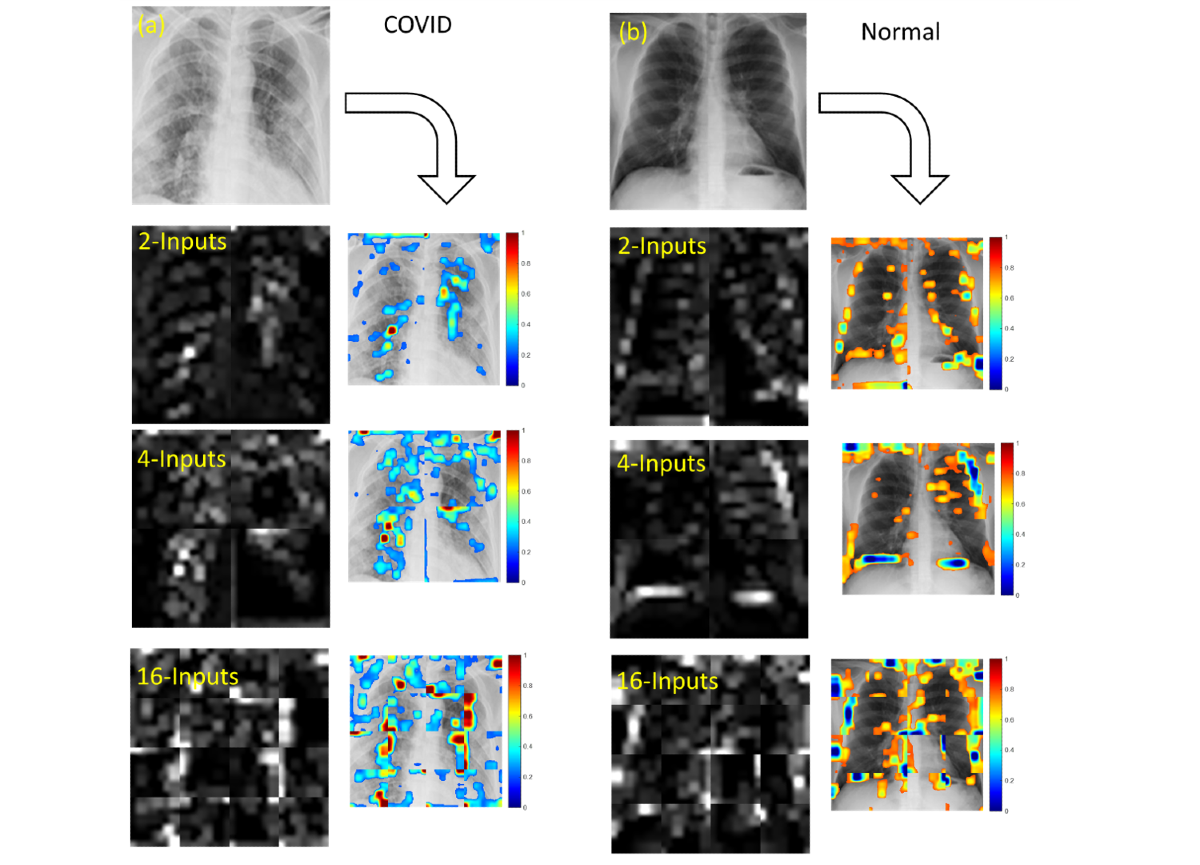
Visualization and mapping of convolutional neural network (CNN) features extracted from the strongest activations of the max-pooling layers using multiple-input CNNs with different numbers of inputs and the left- and right-lung region of interest data sets. (a) CNN features of COVID-19 chest X-ray radiographs (CXRs); (b) CNN features of normal CXRs. In color mapping, the red regions indicate COVID-19 features and the blue regions indicate normal features.

Figure 13 provides a visual representation of the CNN features of MI-CNNs for the LR-ROI data sets. In COVID-19 CXRs, most of the strong-intensity pixels (red regions) in the visualized CNN features were distributed in the lung regions. Compared to the left lung, more color mapping was located in the right-lung regions, especially for the 4-input MI-CNN (Figure 13a). In normal CXRs (blue regions), most of the CNN features extracted by MI-CNNs with LR-ROI data sets could be found at the lung edges (Figure 13b).

Compared to the LR-ROI data sets, MI-CNNs had a much lower efficiency for extracting CNN features in the whole-image data sets. Most of the critical features (the strong-intensity pixels) were found in the nonlung regions (Figure S6a in Multimedia Appendix 1). In the normal CXRs, several of the CNN features (the blue regions in the color map) were distributed at the lung edges; however, some features were found in the nonlung regions (Figure S6b in Multimedia Appendix 1).

## Discussion

### Principal Findings

Currently, the COVID-19 pandemic is still deeply impacting the world and threatening many people’s lives [1]. CXR remains one of the most commonly used imaging modalities for diagnosing COVID-19 owing to its advantages of low radiation, lack of side effects, economic feasibility, and moderate sensitivity [11-16,38]. In this study, we developed a novel MI-CNN method to classify COVID-19 and normal CXR images. In the whole-image CXR training, the results showed that MI-CNNs exhibited good efficiency for COVID-19 classification with high training accuracy and testing performance (over 95% accuracy, sensitivity, specificity, and precision). Compared to the 1-input CNN, the MI-CNNs had higher efficiency in recognizing COVID-19 CXRs, especially for the 4- and 16-input MI-CNNs, with testing accuracy over 97%. MI-CNNs also showed higher accuracy than the 1-input CNN from the beginning of training progress. Therefore, splitting the CXRs into different regions could improve the efficiency in classifying COVID-19 and normal CXRs. Regarding the learning rates, using a smaller learning rate (0.001) could indeed help to increase the accuracy, sensitivity, specificity, and precision from those of 1-input CNN models, by approximately 2%-3%. However, the learning rate had almost no effect for the MI-CNN models (2-input and 4-input MI-CNNs, as shown in Figure S7 of Multimedia Appendix 1).

In this study, CXRs were evenly segmented into different regions, and each image segmentation could individually serve as one of the MI-CNN inputs. Through assessment of the image percentage of the accurately classified CXRs in the testing data sets, the contributions of each region could be evaluated; in particular, more detailed contributions of each region could be screened with more MI-CNN inputs (eg, 16-inputs MI-CNN). According to our results, some CNN features could allow the network to determine the correct classification, whereas some image features may cause serious misjudgment [34]. Although the whole-image data sets could obtain higher accuracy for COVID-19 identification than LR-ROI data sets, some of the contributions were from the nonlung regions. For instance, in the 16-inputs MI-CNN, R1 regions had accurate contributions for COVID-19 classification, but the nonlung region R16 also had remarkable contributions for COVID-19 classification. Therefore, if using medical images as single inputs, not all regions will provide the correct contributions for classifying COVID-19 CXRs. Some of the nonlung regions may give noticeable contributions falsely.

Moreover, extraction of the lung regions (LR-ROI data sets) could greatly help to extract the critical regions for COVID-19 classification. Compared to the whole-image data sets, the critical regions could be found at R1, R2, R3, R5, R9, and R13 in the LR-ROI data sets. These regions significantly contributed to accurately classifying COVID-19 CXRs (eg, R2, R9, and R13 contributed up to approximately 65% of accurately classified COVID-19 CXRs), but had no significant contribution to the normal CXRs. In comparison, R6, R10, R12, R14, and R16 were found in normal CXRs. Among them, R6, R10, and R12 contributed to over 80% of the accurately classified normal CXRs, whereas these regions had almost no contribution to COVID-19 classification.

In addition, right-lung regions had a higher contribution in the classification of COVID-19 than left-lung regions. By using the 16-inputs MI-CNN, more critical regions were screened in the right lung. Moreover, the sensitivity of the right-lung data sets with the 1-input CNN (approximately 94%, as shown in Figure 8b) was higher than that of the left-lung data sets (approximately 91%), which also indicates that right-lung regions tend to be more efficient in the classification of COVID-19 CXRs. By contrast, excluding the LR-ROI with the 16-input MI-CNN (Figure 12b), most of the critical regions related to normal CXRs could be found in the left-lung regions, which was further demonstrated through the higher precision (94%) in the left-lung ROI (Figure 8c). Based on their distributions, more critical regions related to COVID-19 were also found in the right-lung regions. In contrast, more regions related to normal CXRs were found in the left lung, especially for the 16-inputs MI-CNN with LR-ROI data sets (Figure S4b in Multimedia Appendix 1).

Finally, from the visualized CNN features, MI-CNNs still had better feature extractions than the 1-input CNN. For the CXRs in cases of severe COVID-19 in the whole-image data sets, CNN features extracted by the 1-input CNN were mainly distributed in the lung regions (Figure S5 in Multimedia Appendix 1). However, for the CRXs in cases of mild COVID-19, most of the CNN features were found at the lung edges (not within lung regions). Therefore, using the 1-input CNN to extract CNN features would be highly impacted by the quality of the whole-image CXRs. However, LR-ROI data sets could provide higher accuracy for COVID-19 classification than the whole-image data sets. Most of the critical features related to COVID-19 classification were distributed within the lung regions. This point is consistent with the evaluations of the critical regions, in which LR-ROI data sets could give more accurate COVID-19 classifications than the whole-image data sets. MI-CNNs tended to identify the normal CXRs from the edges of the lung regions in the LR-ROI data sets. Most of the strong-intensity regions in the visualized CNN features were distributed around the lung edges.

### Comparison With Prior Work

Although previous studies reported that deep-learning methods could achieve very high accuracy in classifying COVID-19 CXRs or CT scans, most of these analyses were based on whole images as the single input, and the specific regions that contribute to the successful classification of COVID-19 have barely been explored [8,9,13,15,16,18,20,26-29]. Compared to the single-input CNN model, MI-CNNs will provide more CNN features for the classification, which could improve the accuracy of the entire system [33,34]. However, most previous studies focused on whole images with different formats as the CNN inputs [33,34], and few studies attempted to segment a medical image into different regions as the CNN inputs and evaluate their contributions to the final classification of disease images. Therefore, our MI-CNNs could independently extract features from different regions; more importantly, the contribution of each region could be evaluated through the MI-CNN models.

Moreover, compared to the traditional CNN models GoogLeNet and ResNet_50, our proposed 1-input model could achieve similar performance with the same data set (whole CXRs) and learning rate (0.001). Our model also required much less time (only approximately 14 minutes for each training), whereas GoogLeNet and ResNet_50 needed respectively more than 130 minutes and 300 minutes, representing an increase of 10 times and 20 times than required with our models.

### Limitations

Although the MI-CNNs could achieve good efficiency in the classification of COVID-19 CXRs and screen the critical regions and features related to COVID-19, there are still several major limitations of this study. First, the size of the COVID-19 and normal data set is still small, and more CXRs are required to further test the reliability of our MI-CNNs. Second, the feature visualization exhibits relatively low efficiency. Other algorithms such as GRAD can be used to better map the critical features to the original CXR. Third, all of the MI-CNN models used the same structures of convolutional layers for all CXR regions. More complicated structures could be further explored in the future. For example, different regions could use different convolutional designs, such as the lung boundary with fewer convolutional layers and lung regions with more convolutional layers. Finally, the severity of COVID-19 cannot currently be evaluated with MI-CNN models, especially from the critical features. Finally, more parameters (eg, image resolution) could be used to better evaluate the accuracy and performance of MI-CNNs.

### Conclusions

In summary, each MI-CNN input could individually process only one part of CXRs, which contributes to the highly efficient classification of the COVID-19 CXRs. In the whole-image data sets, MI-CNNs could achieve better classification efficiency (over 95% accuracy, sensitivity, specificity, and precision) than the 1-input CNN. In addition, the performance of MI-CNNs increased with the number of inputs, especially for the 4- and 16-input MI-CNNs with over 97% accuracy. In the LR-ROI data sets, the MI-CNNs showed an approximate 4% decrease in the classification of COVID-19 CXRs compared to the whole-image data sets. Some nonlung regions (eg, R16) had positive contributions to COVID-19 classification (also shown in the visualized CNN features), which fraudulently increased the higher performance in the whole-image data sets. Therefore, compared to the whole-image data sets, LR-ROI data sets could provide a more accurate evaluation for the contribution of each region, as well as the extraction of CNN features.

From the analysis of the contributions of critical regions in the testing data sets, the right lung had a greater contribution to the classification of COVID-19 CXRs. However, the left-lung regions had a greater contribution to classifying normal CXRs. From LR-ROI data sets, MI-CNNs were sensitive to the lung edges and found more important features distributed around the lung edges in normal CXRs. For COVID-19 CXRs, visualized CNN features were primarily distributed within the lung regions (especially in the 16-inputs MI-CNN).

In conclusion, MI-CNNs have excellent efficiency in classifying COVID-19 CXRs. More MI-CNN inputs usually result in better classification efficiency. Our method could assist radiologists in automatically screening the regions playing critical roles in the classification of COVID-19 from CXRs.

## Appendix

Multimedia Appendix 1 Supporting information; Figures S1-S8.

## Abbreviations

AUC: area under the receiver operating characteristic curve
CNN: convolutional neural network
CT: computed tomography
CXR: chest X-ray radiography
FC: Fully connected layer
LR-ROI: left- and right-lung region of interest
MI-CNN: multiple-inputs convolutional neural network
PCR: polymerase chain reaction
ReLu: rectified linear unit
ROC: receiver operating characteristic
ROI: region of interest
RT-PCR: reverse transcription real-time polymerase chain reaction
WHO: World Health Organization
