# Supplementary material for: Multiple-Inputs Convolutional Neural Network for COVID-19 Classification and Critical Region Screening From Chest X-ray Radiographs: Model Development and Performance Evaluation
Source: JMIR Bioinform Biotechnol. 2022 Oct 4;3(1):e36660. doi: 10.2196/36660 (PMC9578294; doi:10.2196/36660)
Supplement: Multimedia Appendix 1 [file bioinform_v3i1e36660_app1.docx]

***Multimedia Appendix 1***


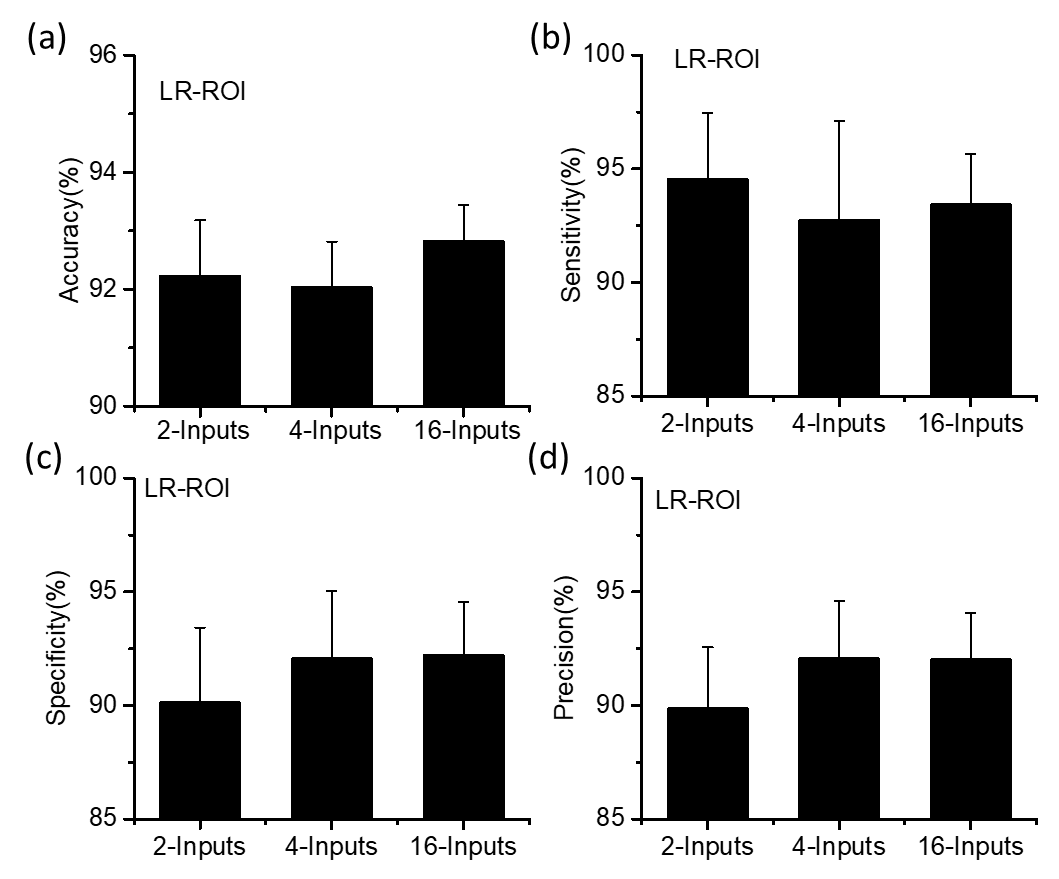


Fig. S1 (a) Accuracy, (b) sensitivity, (c) specificity, (d) precision of the of 2-Inputs, 4-Inputs, and 16-Inputs MI-CNNs with LR-ROI datasets.


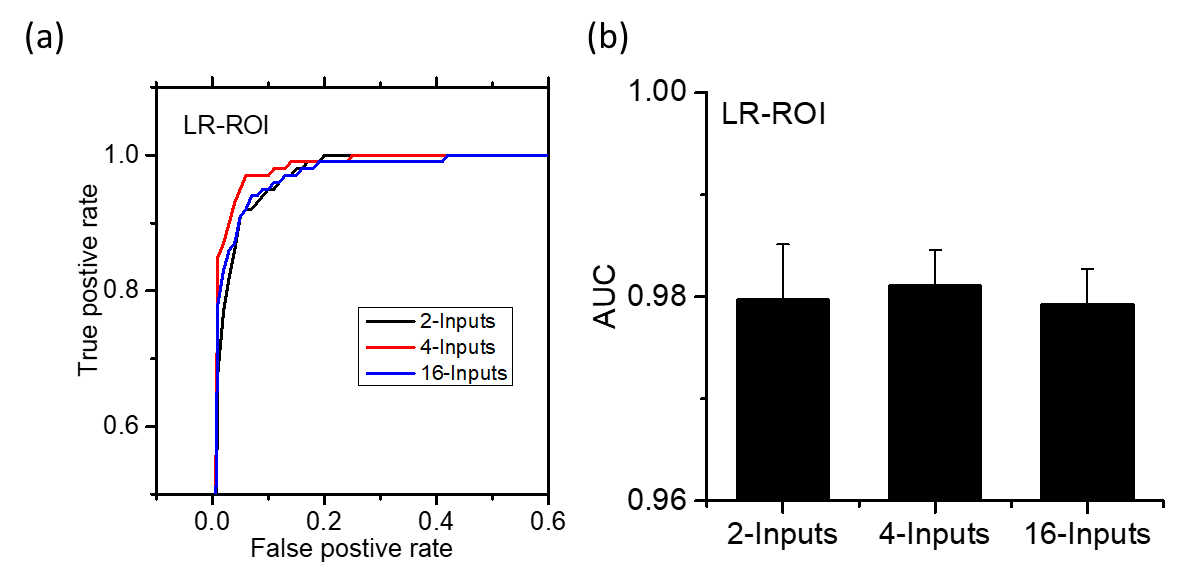


Fig. S2 Receiver operating characteristic curves (a) and AUC (b) of 2- Inputs, 4- Inputs, and 16- Inputs MI-CNNs with LR-ROI datasets.


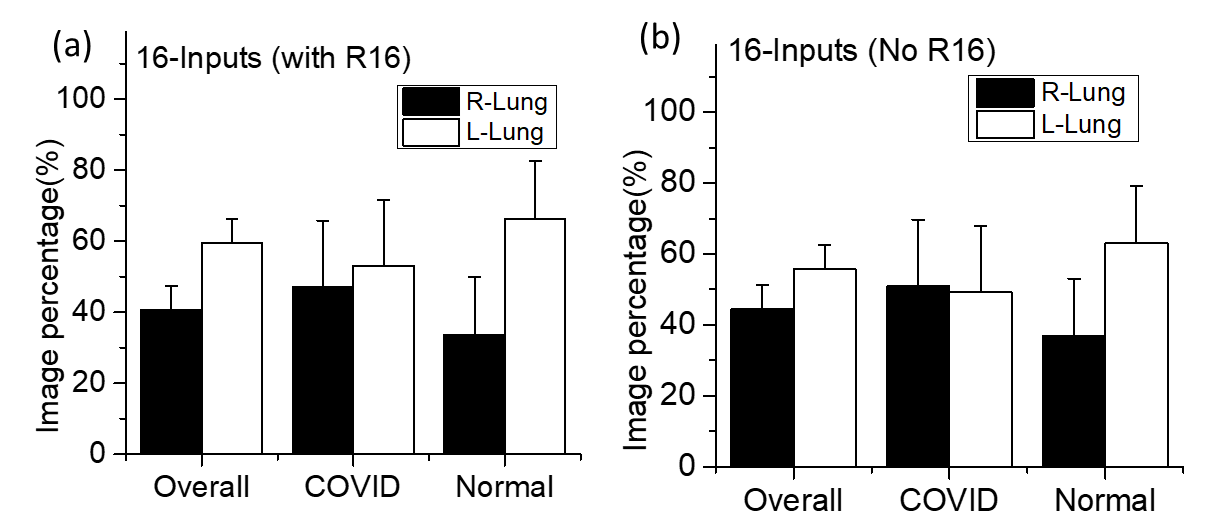


Fig. S3 The correction of the L-Lung and R-Lung contribution for the classification of COVID CXRs by removing the R6 outputs. (a) L-Lung and R-Lung contribution with R16 regions; (b) L-Lung and R-Lung contribution without R16 regions.


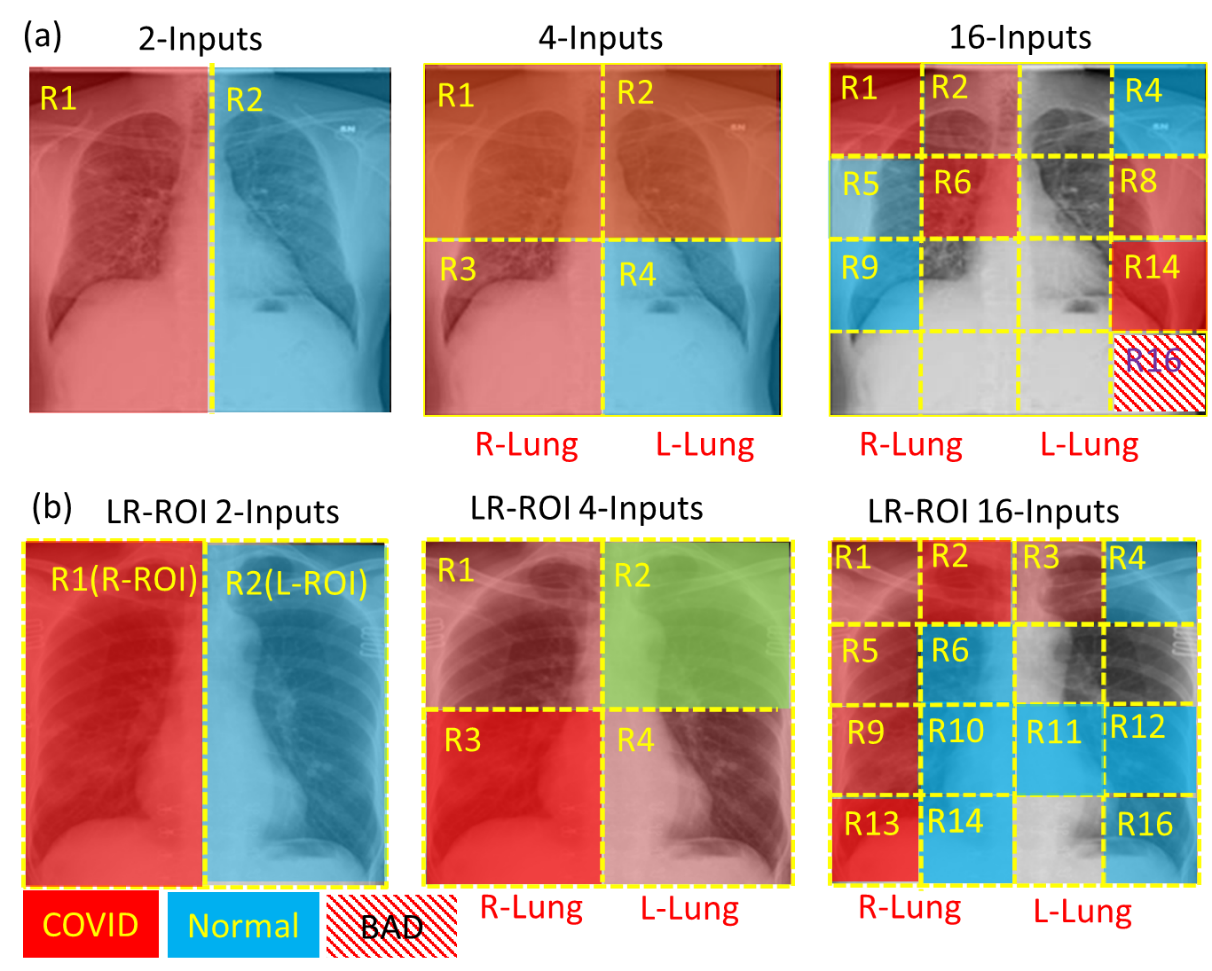


Fig. S4 The distributions of the critical region contribution to the COVID classification in the CXR images under the whole-image and LR-ROI datasets. (a) The whole-image datasets; (b) LR-ROI datasets.

*
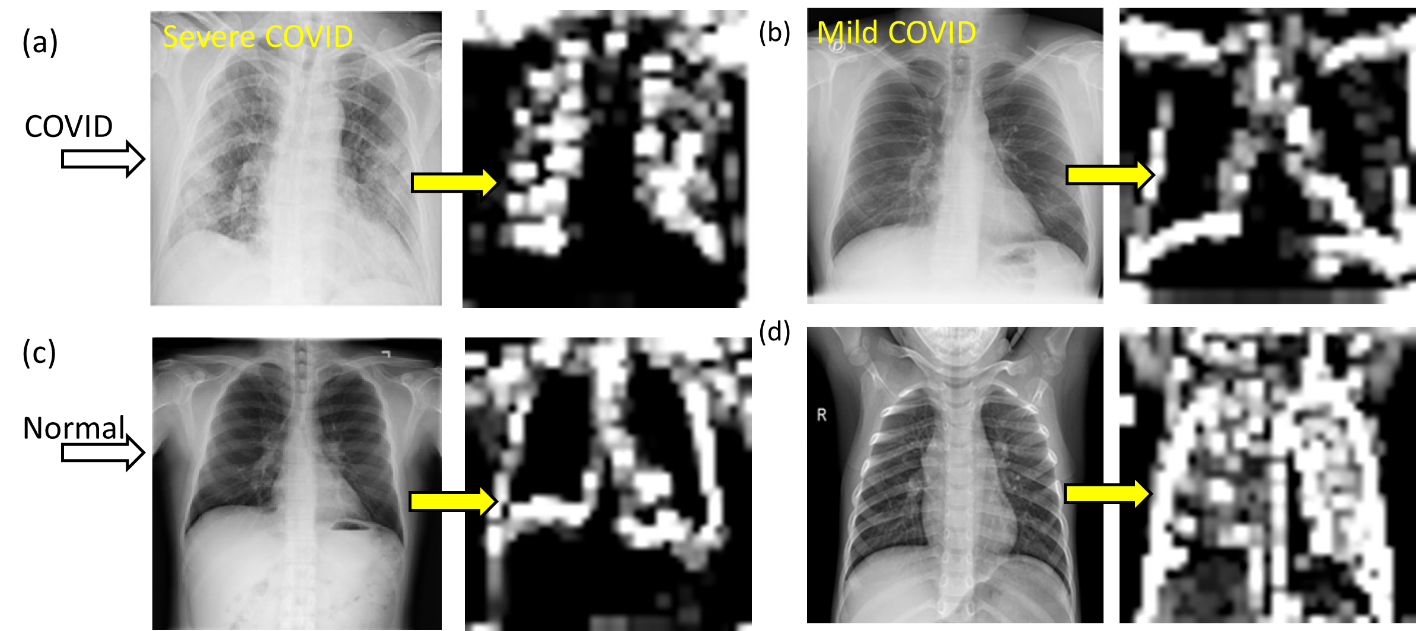
*

Fig. S5. Visualization and mapping of CNN features of severe and mild COVID, as well as Normal CXRs extracted from the strongest activations of max-pooling layers under 1-Input MI-CNNs by using the whole-image datasets. (a) severe and (b) mild COVID CNN features under the whole-image datasets; (c) and (d) Normal CNN features in the whole-image datasets.

Fig. S5 showed the typical CNN features of COVID and Normal CXRs that were extracted from the max-pooling layers of 1-Input MI-CNNs. From severe COVID CXRs in the whole-image datasets (Fig. S5a), both the left and right lung regions of COVID CXRs had very strong pixel intensity in the visualized CNN features, which may play important roles in finding the features related to COVID diseases. In the Normal CXRs, For the Normal CXRs with clear lung regions, the visualized CNN features indicate that the single input MI-CNN classified the Normal CXRs through the edges of the lung regions, which most of the pixels with the strong intensity in visualized CNN features were from the edges of the lung regions (Fig. S5c).

However, in the mild COVID CXRs (Fig. S5b), its visualized CNN features were similar to Normal CXRs (Fig. S5c) in that most of the important features (the strong-intensity pixels) were found at the lung edges. When the other Normal CXRs with stronger rib regions, most of the lung regions were also classified as important features in the visualized CNN features, but the lung edges were still considered as the important features for the Normal classification (Fig. S5d).


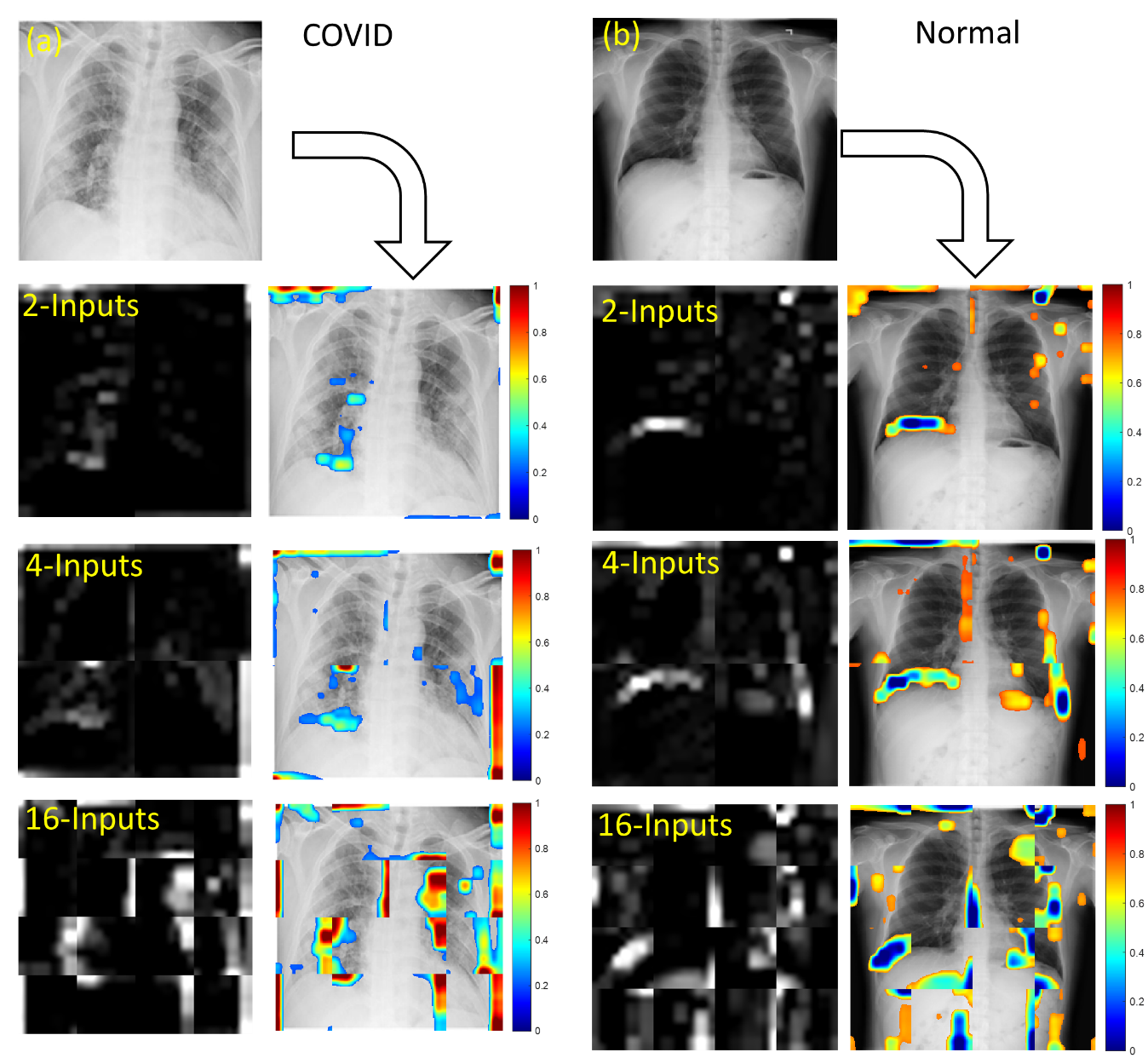


Fig. S6 Visualization and mapping of CNN features extracted from the strongest activations of the max-pooling layers under by using different-inputs MI-CNNs with the whole-image datasets. (a) CNN features of COVID CXRs; (b) CNN features of Normal CXRs. In color mapping, the red regions towards COVID features and the blue regions towards Normal features.


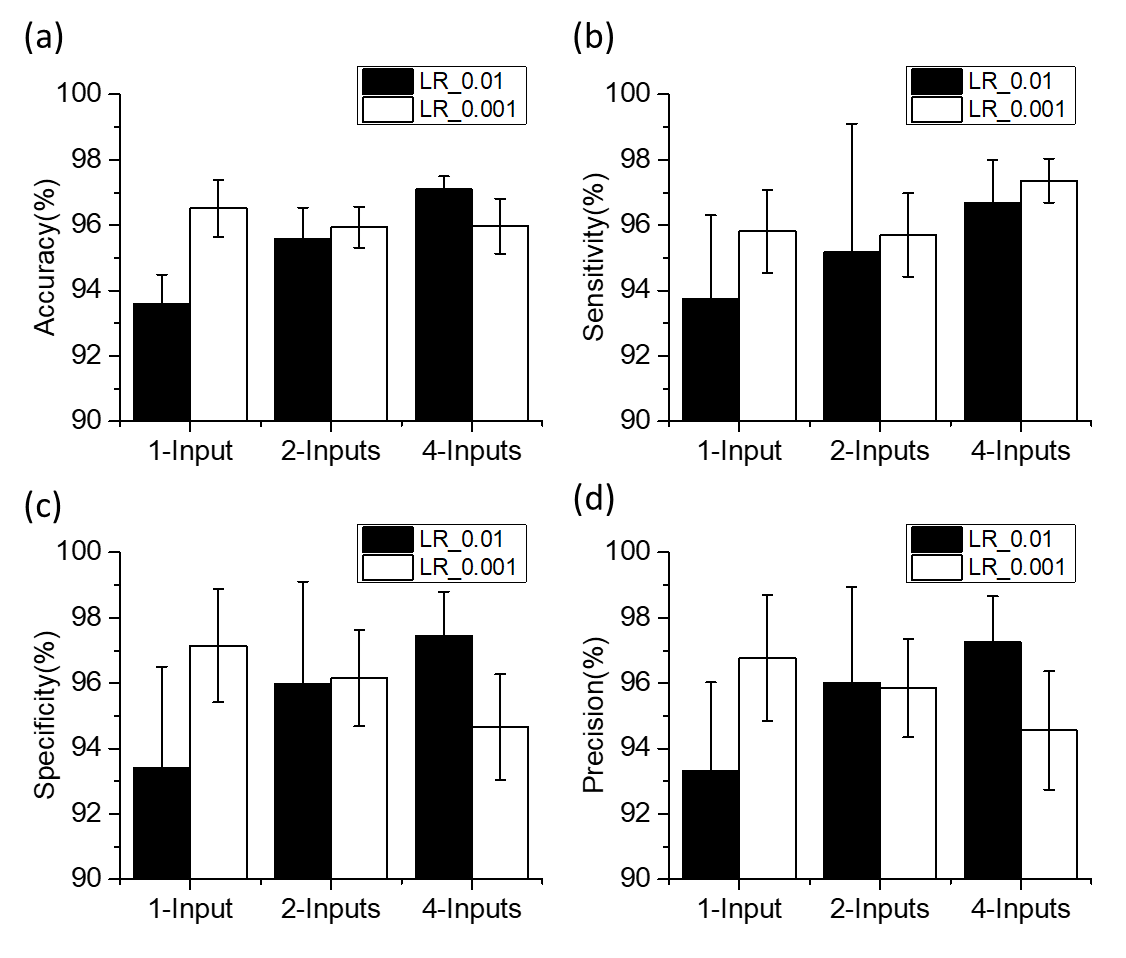


Fig. S7 Accuracy (a), sensitivity (b), specificity(c), and precision (d) of 1-Input, 2-Input and 4-InputS MI-CNNs under learning rates of 0.01 and 0.001. 16-Inputs MI-CNN was overflow under learning rate of 0.001.


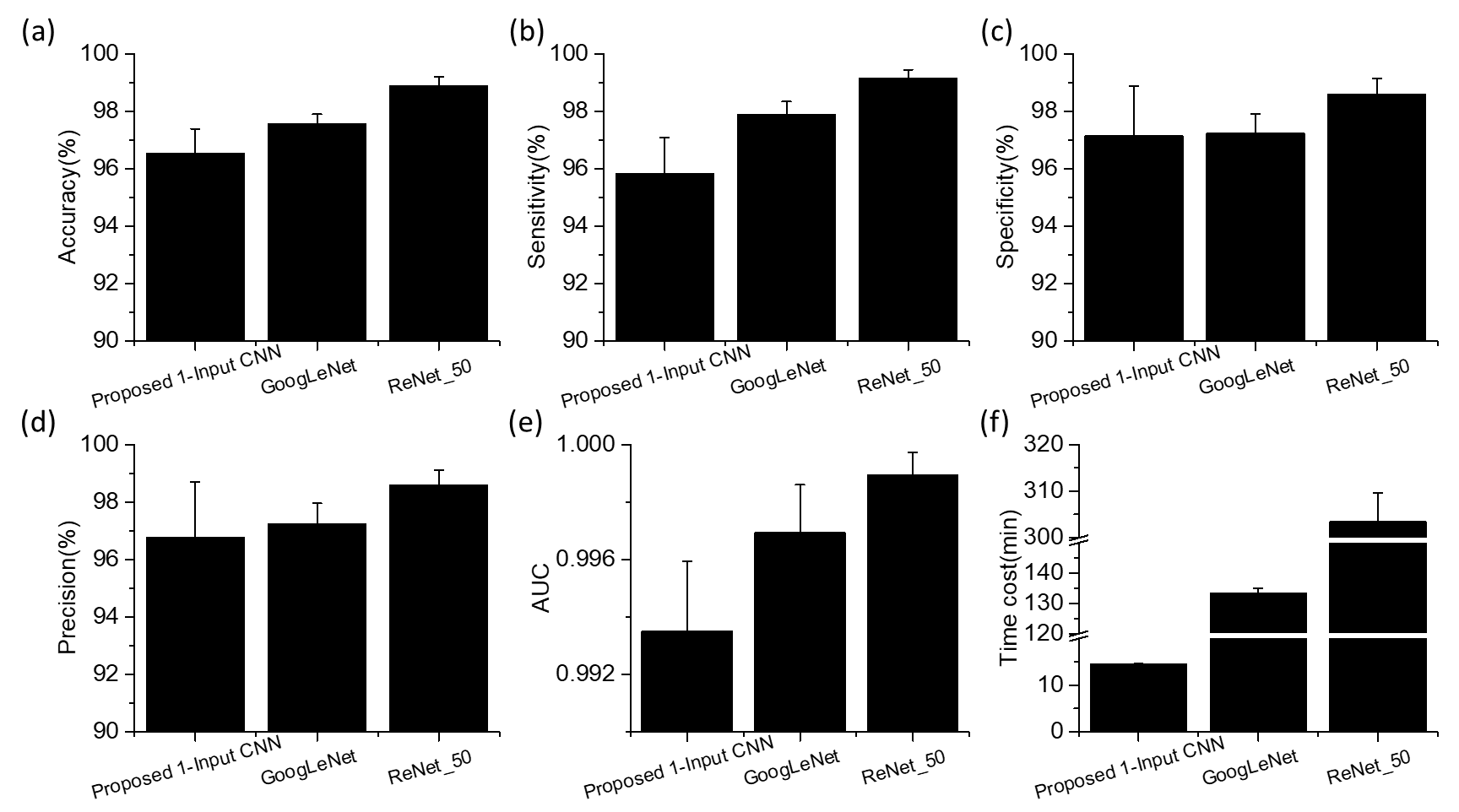


Fig. S8 Compared the proposed CNN (1-Input) with traditional CNN models: GoogLeNet and ResNet_50. (a) Accuracy, (b) sensitivity, (c) specificity, (d) precision, (e) AUC, and (f) time cost for each training.
